# Impact of a Teledermatology-Based Referral Model on Melanoma Diagnostic Pathways and Clinicopathologic Features: A Retrospective Comparative Study Between Face-to-Face Consultation (2019) and Teledermatology (2022) in a Tertiary Hospital

**DOI:** 10.3390/jcm15010267

**Published:** 2025-12-29

**Authors:** Marta Cebolla-Verdugo, Husein Husein El-Ahmed, Francisco Manuel Ramos-Pleguezuelos, Ricardo Ruiz-Villaverde

**Affiliations:** 1 Department of Dermatology, Hospital Universitario San Cecilio, 18016 Granada, Spain; marta.cebolla.sspa@juntadeandalucia.es; 2 Instituto de Investigación Biosanitaria de Granada (ibs.GRANADA), 18012 Granada, Spain; husein.husein.sspa@juntadeandalucia.es; 3 Department of Dermatology, Hospital de Baza, 18016 Granada, Spain; 4 Department of Pathology, Hospital Universitario San Cecilio, 18016 Granada, Spain

**Keywords:** melanoma, teledermatology, Breslow thickness, histologic regression, immunosuppression, multivariable logistic regression

## Abstract

**Background/Objectives:** Teledermatology has transformed access to dermatologic care, yet its association with melanoma prognostic parameters and diagnostic pathways in tertiary settings remains incompletely characterized. To compare the clinicopathologic profile of melanomas diagnosed under face-to-face consultation (2019) versus teledermatology-based referral (teleconsultation) (2022). **Methods:** A retrospective observational study comparing two patient cohorts: those diagnosed with melanoma via in-person consultation in 2019, and those diagnosed through teleconsultation in 2022. These years were selected to reflect the structural shift in care delivery models before and after the COVID-19 pandemic, during which teledermatology was formally implemented. Sociodemographic, clinical, and histopathological variables were collected. A multivariable logistic regression model assessed variables associated with being diagnosed in the 2022 teledermatology cohort versus the 2019 face-to-face cohort. Statistical analyses were performed using R (v. 4.4.3). **Results:** A total of 151 patients were included (89 in-person in 2019, 62 via teleconsultation in 2022). Multivariable analysis identified three variables independently associated with being diagnosed via teleconsultation. Increasing Breslow thickness was inversely associated with teleconsultation diagnosis (OR 0.60 per 1 mm increase; 95% CI 0.40–0.91; *p*= 0.017). Similarly, the presence of histologic regression (OR 0.28; 95% CI 0.09–0.90; *p* = 0.032) and immunosuppression (OR 0.08; 95% CI 0.008–0.86; *p* = 0.037) were inversely associated with teleconsultation diagnosis. No significant associations were found for sex, age, tumor location, ulceration, mitosis, or clinical stage. **Conclusions:** In this retrospective single-center comparison of two care models, melanomas diagnosed through teleconsultation in 2022 were associated with a more favorable clinicopathologic profile at diagnosis than those diagnosed via face-to-face consultation in 2019. These findings support the role of teledermatology-based referral pathways in facilitating timely melanoma assessment, although causal inference is limited by the observational design.

## 1. Introduction

Melanoma is the leading cause of skin-cancer-related mortality worldwide [1,2], accounting for over 331,000 new cases and 58,000 melanoma-attributable deaths globally in 2022 alone [1]. Its incidence continues to rise, particularly among fair-skinned populations [3], and has been strongly linked to greater ultraviolet (UV) radiation exposure, both from sun-related behaviors and artificial tanning devices [4,5]. In southern Europe, including Spain, incidence rates remain on the rise, particularly for early-stage melanomas such as AJCC stage 0 and stage I melanoma (8th edition) [6]. Survival has improved over recent decades, likely driven by earlier detection and advances in systemic therapies; however, disparities in access and outcomes persist across geographic and demographic lines [6,7].

Breslow thickness remains the most robust prognostic marker for melanoma, critically influencing both survival outcomes and treatment costs, which escalate dramatically with advanced-stage disease [8]. In this context, teledermatology has emerged as a tool to support timely assessment and referral of suspicious pigmented lesions. Several studies have reported an association with thinner Breslow thickness at diagnosis and a higher proportion of early-stage melanomas (stages 0 and I) [9]. Recent national data from Spain further support its role in improving diagnostic timelines and access to care, particularly for malignant and premalignant lesions [10,11,12].

Despite this promise, implementation of teledermatology systems remains heterogeneous, and evidence from post-pandemic tertiary care settings is limited. Although early reports highlighted positive physician perceptions and organizational challenges during the COVID-19 pandemic [13], few studies have rigorously evaluated the clinical impact of teledermatology in specialized care [14]. Moreover, most published data stem from primary care settings, and there is a notable scarcity of multivariable analyses assessing clinicopathologic predictors such as Breslow thickness or clinical staging across consultation modalities [9]. Therefore, evidence from tertiary post-pandemic settings remains needed to inform resource allocation and equitable access to specialized dermatologic care.

This study aimed to compare the clinicopathologic features of melanomas diagnosed via teleconsultation and in-person consultation in a tertiary care center across two periods reflecting different care delivery models (2019 vs. 2022), with a particular focus on prognostic indicators such as Breslow thickness and clinical stage.

## 2. Methods

### 2.1. Study Design and Population

We conducted a retrospective observational study at Hospital Universitario San Cecilio in Granada, Spain. The hospital serves a reference population of approximately 483,000 inhabitants. The study population comprised two cohorts of adult patients diagnosed with cutaneous melanoma: one via in-person dermatologic consultation in 2019 and another through teleconsultation in 2022. These years were selected to represent two structurally distinct care models implemented before and after the COVID-19 pandemic.

### 2.2. Referral Pathways and Care Models

In 2019, patients with suspicious skin lesions were referred by primary care physicians to the Dermatology outpatient clinic through the standard referral pathway, where diagnosis was established after in-person assessment and subsequent excisional biopsy when indicated. The mean interval between referral and dermatologic evaluation during this period was 55.8 days.

In 2022, suspected lesions from primary care were channelled through a store-and-forward teledermatology system as the routine access route for dermatology consultation. Primary care physicians submitted standardized clinical photographs and, when available, dermoscopic images; submissions were asynchronously reviewed by board-certified dermatologists, who issued an assessment within 24 h. When melanoma was suspected, patients were prioritised for expedited in-person assessment and biopsy scheduling (typically within 48 h after the teledermatology assessment). Dermoscopic imaging resources were not uniformly available across referring primary care centres, and therefore dermoscopic images were not consistently included. Patients diagnosed through internal hospital follow-up visits or referred from other institutions were not included in the teledermatology cohort, as the study defined cohorts based on the entry route from primary care (teledermatology in 2022 vs. conventional referral in 2019).

For the present study, the 2019 cohort comprised melanomas diagnosed through the face-to-face pathway, whereas the 2022 cohort comprised melanomas diagnosed through the teleconsultation pathway; patients did not self-refer to teleconsultation.

All patients were managed within the same tertiary care hospital system.

### 2.3. Inclusion and Exclusion Criteria

Eligible participants were adults (age ≥ 18 years) with histologically confirmed melanoma diagnosed through excisional biopsy. Patients diagnosed via punch or incisional biopsy, those managed surgically at other institutions, or those with missing essential data were excluded.

### 2.4. Data Collection and Variables

Clinical and histopathologic data were extracted from electronic medical records (Diraya^®^ system, v 4.38.0, Spain) and pathology reports. Sociodemographic variables included age, sex, and consultation modality. Clinical variables encompassed tumor location, skin phototype (Fitzpatrick scale), history of chronic or intermittent sun exposure, childhood sunburns, and immunosuppression status. Inmunosupression was defined as solid-organ transplantation, hematologic malignancy, HIV infection, or ongoing systemic immunosuppressive therapy. Histologic variables included Breslow thickness (mm), ulceration, mitotic activity, Clark level, histologic regression, lymphovascular and perineural invasion, and clinical stage according to the AJCC 8th edition. Sentinel lymph node biopsy results and presence of metastases were also recorded.

### 2.5. Statistical Analysis

Continuous variables were summarized using means ± standard deviation (SD) and assessed for normality using the Shapiro–Wilk test. Categorical variables were expressed as frequencies and percentages. Between-group comparisons were conducted using Welch’s *t*-test for normally distributed continuous variables and the Mann–Whitney U test for non-parametric data. Pearson’s chi-square test or Fisher’s exact test were used for categorical variables.

A multivariable logistic regression model was fitted to examine the association between clinicopathologic variables and the consultation modality (teleconsultation vs. face-to-face) across the two study periods. All relevant demographic, clinical, and histopathologic variables were entered simultaneously into the model. Results are reported as regression coefficients, odds ratios (ORs) with 95% confidence intervals (CIs), and *p*-values. A two-sided *p*-value < 0.05 was considered statistically significant.

All statistical analyses were performed using R software (version 4.4.3). Graphical representations were generated using the ggplot2 packag version 4.0.1.

This study is reported in accordance with the STROBE (Strengthening the Reporting of Observational Studies in Epidemiology) guidelines (Supplementary Material File S1).

### 2.6. Ethical Considerations

This study was approved by the Institutional Review Board of Hospital Universitario San Cecilio (DERM_HUSC_2024_002). No experimental interventions were conducted. Patient confidentiality was maintained throughout in accordance with the European General Data Protection Regulation (GDPR). The study adhered to the ethical principles outlined in the Declaration of Helsinki.

## 3. Results

### 3.1. Study Population

The study population comprised 151 patients with histologically confirmed cutaneous melanoma: 89 diagnosed during the face-to-face period in 2019 (58.9%) and 62 diagnosed during the teledermatology period in 2022 (41.1%). The mean age was comparable between groups (61.14 ± 16.75 years in 2019 vs. 59.08 ± 16.01 in 2022), and no significant differences were observed in sex distribution (Table 1). Among patients diagnosed during the teledermatology period (2022), dermoscopic images were available in 24 of 62 cases (38.7%).

### 3.2. Baseline Demographic and Clinical Characteristics

Baseline demographic characteristics, personal and family history, and melanoma risk factors are summarized in Table 1.

No relevant differences were observed between periods regarding age, sex, Fitzpatrick phototype, eye color, hair color, personal or family history of melanoma, or history of non-melanoma skin cancer.

Immunosupression was less frequent in the teledermatology period (1.6% vs. 9.0%). Patterns of chronic and intermittent sun exposure, as well as childhood sunburns, were broadly comparable between groups.

### 3.3. Tumor Characteristics

Anatomic site distribution did not differ substantially between periods, with the lower limbs and trunk being the most common locations in both cohorts.

Histopathologic subtypes showed similar distributions overall, with superficial spreading melanoma, nodular melanoma, and lentigo maligna melanoma accounting for the majority of cases. Desmoplastic melanoma was observed only in the 2019 cohort, reflecting its low overall frequency.

### 3.4. Histopathologic Features

Melanomas diagnosed during the teledermatology period showed a lower mean Breslow thickness compared with those diagnosed during the face-to-face period (1.20 ± 1.11 mm vs. 1.44 ± 1.54 mm). Histologic regression was less frequently observed in the teledermatology cohort (29.0% vs. 37.1%). No relevant differences were found between periods regarding ulcerations, mitotic activity, Clark level, lymphovascular invasion, perineural invasion, or tumor size.

### 3.5. Staging and Treatment

The proportion of melanoma in situ and AJCC stage I disease was numerically higher during the teledermatology period, whereas more advanced stages were slightly more frequent during the face-to-face period, although these differences did not reach statistical significance after adjustment. Rates of sentinel lymph node biopsy, sentinel node positivity, and adjuvant treatment were comparable between cohorts.

Multivariable analysis identified three variables independently associated with being diagnosed via teleconsultation. Increasing Breslow thickness was inversely associated with teleconsultation diagnosis (OR 0.60 per 1 mm increase; 95% CI 0.40–0.91; *p* = 0.017). Similarly, the presence of histologic regression (OR 0.28; 95% CI 0.09–0.90; *p* = 0.032) and immunosuppression (OR 0.08; 95% CI 0.008–0.86; *p* = 0.037) were inversely associated with teleconsultation diagnosis.

**Figure 1:**
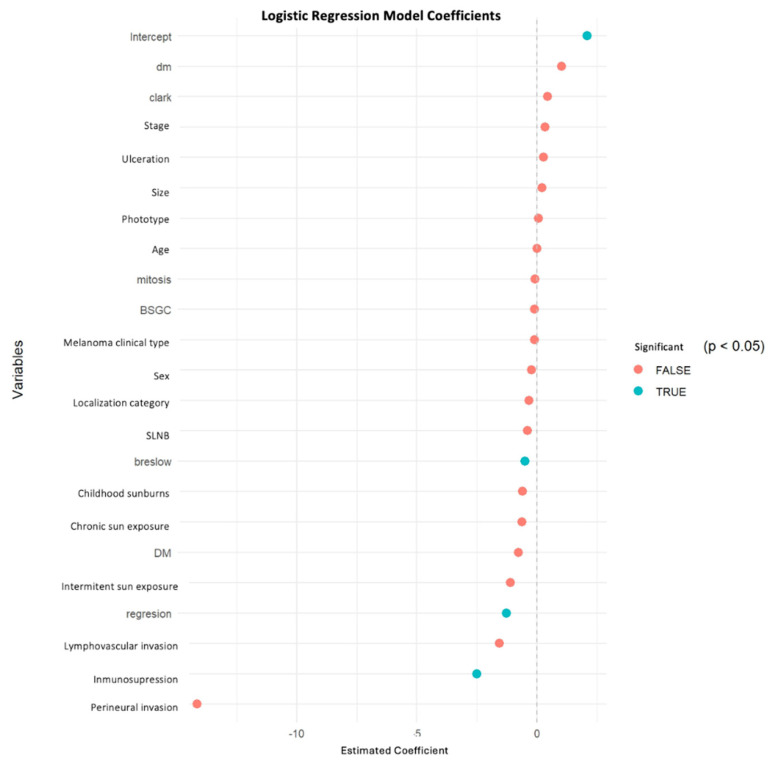
Multivariable Logistic Regression Coefficients comparing melanomas diagnosed during the teledermatology period (2022) versus the face-to-face period (2019). Statistically significant variables (*p* < 0.05) are highlighted in turquoise, while non-significant variables are shown in red. Positive coefficients indicate a higher likelihood of diagnosis through teleconsultation, whereas negative coefficients indicate a lower likelihood of teleconsultation.

**Figure 2:**
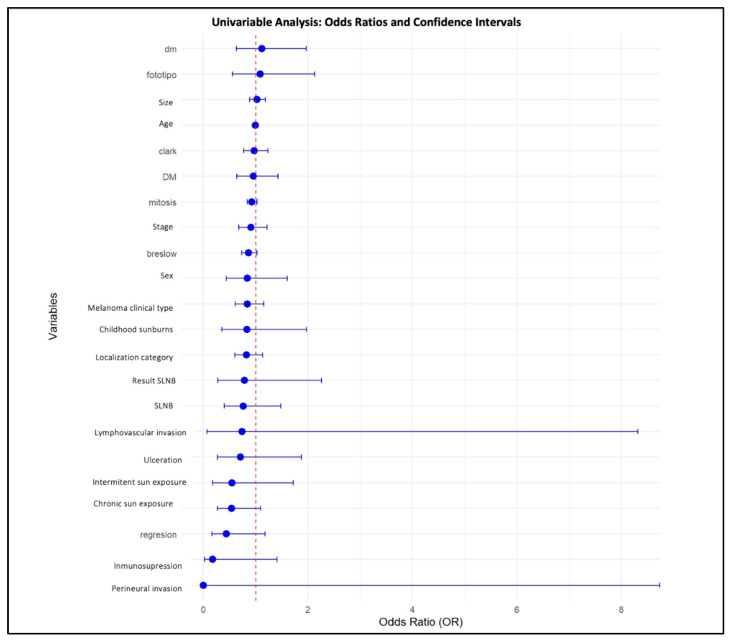
Univariable Logistic Regression Analysis comparing the teledermatology period (2022) with the face-to-face period (2019). Odds ratios (ORs) with 95% confidence intervals are shown. An OR > 1 indicates higher odds of diagnosis during the teledermatology period, whereas an OR < 1 indicates lower odds. Variables whose confidence intervals do not cross OR = 1 are statistically significant (*p* < 0.05).

Other variables, including sex, age, ulceration, mitotic rate, tumor location, and clinical stage, were not significantly associated with the consultation modality after multivariable adjustment. These results are summarized in Table 2, and graphically represented in Figure 1 (multivariable model coefficients) and Figure 2 (univariate odds ratios and confidence intervals).

## 4. Discussion

In this retrospective comparison of two non-concurrent care models (face-to-face consultation in 2019 vs. store-and-forward teleconsultation in 2022), melanomas diagnosed during the teleconsultation period showed a more favorable histopathologic profile, with lower Breslow thickness and lower frequency of histologic regression. In multivariable analysis, increasing Breslow thickness, the presence of regression, and immunosuppression were inversely associated with being diagnosed during the teleconsultation period. These findings should be interpreted as between-period differences in case mix and diagnostic pathways.

Importantly, these findings should not be interpreted as evidence that teledermatology directly influences melanoma biology or intrinsically leads to the diagnosis of thinner or lower-risk tumors. Rather, the observed differences are more appropriately understood as reflecting variations in case mix and referral pathways across two structurally distinct care models. In this context, teledermatology primarily functions as a triage and prioritization mechanism, facilitating expedited specialist review of selected lesions, rather than as an independent diagnostic modifier. This interpretation is consistent with prior reports emphasizing the organizational utility of teledermatology while cautioning against overstatement of its diagnostic impact [15].

A clinically plausible explanation is that lesions with an obvious malignant phenotype—often corresponding to thicker tumors and, in some cases, complex histopathologic features—may have been more represented in the conventional in-person pathway, whereas teleconsultation may be particularly useful for triaging equivocal pigmented lesions and accelerating specialist review and management. This interpretation is consistent with the literature comparing conventional referral and teledermoscopy. In a Swedish setting, Schultz et al. [16] reported a higher proportion of invasive melanomas and greater Breslow thickness in the traditionally referred group, while melanoma in situ was more frequent among teledermoscopy-triaged cases. Our results align with the direction of these observations and support the concept that teledermatology may contribute to earlier identification of melanoma within structured referral systems.

The observed association between regression and diagnosis during the face-to-face period warrants cautious interpretation. Regression may reflect immune-mediated changes and can coexist with clinical-dermoscopic ambiguity, potentially increasing the perceived need for direct examination in some settings. However, because regression is determined histologically and the two cohorts reflect different organizational models, our data do not allow inference about decision-making at the point of referral. Instead, regression should be viewed as a cohort-level difference in tumor profile.

Immunosuppression was also less frequent among cases diagnosed during the teleconsultation period. This may reflect a combination of clinical caution and follow-up complexity in immunosuppressed patients, who often require closer longitudinal surveillance and may enter care through alternative or expedited routes. Again, given the non-concurrent design, this should be viewed as a cohort-level difference rather than a determinant of pathway allocation.

A key operational feature of the 2022 model was the rapid specialist review inherent to store-and-forward teleconsultation, which can shorten the interval from referral to dermatologic assessment and facilitate timely excision. The average interval from referral to dermatologic evaluation was reduced from 55 days in the in-person model to just 2 days with teleconsultation. This improvement in access timelines has direct implications for patient anxiety, disease progression, and institutional workflow optimization. Similarly, among patients requiring deferred surgical excision under local anesthesia, mean waiting times declined from 16 to 7 days between 2019 and 2022. These improvements are consistent with the clinical standards delineated in the Integrated Care Process for Skin Cancer (Proceso Asistencial Integrado de Cáncer de Piel) established by the Andalusian Regional Health Ministry (Servicio Andaluz de Salud) [17], which advocates for prompt surgical intervention to improve oncologic outcomes and healthcare efficiency.

The structural integration of teledermatology was also catalyzed by the COVID-19 pandemic, which disrupted conventional care pathways and accelerated the adoption of remote diagnostic modalities. Previous studies, such as that by Díaz-Calvillo et al. [18], documented diagnostic delays during the pandemic, accompanied by a surge in the detection of thicker, more invasive melanomas. In contrast, the post-pandemic consolidation of teledermatology services has enabled the re-establishment of efficient diagnostic circuits, particularly for malignant lesions. In a recent multicenter study, the average time from referral to dermatologic consultation decreased to just 2 days for teledermatology compared to 29 days for in-person visits, while the time to treatment was reduced from 101 to 59 days, respectively (Toledo-Pastrana et al., 2025) [10].

Nevertheless, teledermatology performance depends heavily on image quality and, for pigmented lesions, the availability of dermoscopic images. In our teleconsultation cohort, dermoscopic images were available in 38.7% of cases, reflecting heterogeneous access across primary care centers. This limitation may have influenced diagnostic confidence and may have contributed to heterogeneity in documentation and downstream management, as supported by studies [19,20,21] showing that incorporation of teledermoscopy improves diagnostic accuracy and safety for atypical pigmented lesions and melanoma. Therefore, our findings should be interpreted within the context of a real-word implementation where dermoscopy was not uniformly available.

The limited availability of dermoscopic images in the teleconsultation cohort represents a major source of heterogeneity and potential diagnostic uncertainty. Dermoscopy plays a central role in the assessment of melanocytic lesions, particularly for diagnostically challenging entities such as spitzoid melanoma, pigmented epithelioid melanocytoma, and desmoplastic melanoma, which were present in small numbers in our cohort. These lesions are well known to exhibit substantial clinical, dermoscopic, and histopathologic ambiguity and often require in-person examination and expert evaluation. Their inclusion underscores the fact that teledermatology pathways do not replace face-to-face assessment for complex cases and highlights the importance of expanding access to standardized teledermoscopy in primary care to enhance diagnostic confidence and safety [22].

Our results should also be contextualized within the heterogeneous teledermatology literature. While several studies [9] have reported thinner melanomas or higher proportions of early-stage disease in teledermatology pathways, other real-world settings have shown different results. For example, Jaklitsch et al. [23] reported shorter time to evaluation and biopsy after teledermatology, but a higher-risk melanoma profile compared with in-person starters, likely reflecting case selection and referral structure rather than an effect of the modality itself.

Histologic regression should be interpreted with caution in this context. Regression represents a heterogeneous phenomenon that may reflect immune-mediated tumor response, partial lesion involution, or areas of diagnostic uncertainty, rather than a reliable marker of tumor aggressiveness. Because regression is identified histologically after excision, our data do not allow inference regarding its influence on referral decisions or triage allocation. Accordingly, the observed association should be viewed as a cohort-level difference in tumor characteristics rather than as a clinically directive or causal finding.

Although our analysis did not include a formal cost-effectiveness evaluation, the economic implications of timely diagnosis warrant consideration. Prior data from our institution [24] have shown that first-year medical costs for melanoma escalate steeply by stage, ranging from €1689 in stage I to over €88,000 in stage IV. These differences persist over the long term, with maintenance therapies, imaging, and palliative interventions substantially increasing the financial burden on healthcare systems. Therefore, any diagnostic strategy—such as teledermatology—that facilitates earlier-stage detection may indirectly yield substantial economic benefits, even in the absence of direct cost modeling.

Nonetheless, several limitations must be acknowledged. The cohorts represent two distinct time periods with different organization models, and unmeasured confounding, channeling, and documentation bias are possible. We did not quantify the denominator of suspected melanoma referrals that ultimately proved benign in either period, precluding estimation of pathway sensitivity or positive predictive value. Additionally, not all referrals from primary care included standardized dermoscopic imaging. This heterogeneity reflects an asymmetrical distribution of diagnostic resources across the Andalusian Public Health System (SSPA), with access to dermatoscopes varying widely among the 122 primary care centers served by our institution, some of which are located in rural municipalities with fewer than 100 inhabitants. In addition, we did not assess long-term outcomes such as recurrence or melanoma-specific survival.

Finally, this study did not assess long-term oncologic outcomes such as recurrence, upstaging, melanoma-specific survival, or overall survival. Therefore, no conclusions can be drawn regarding the oncologic safety or long-term equivalence of teledermatology-managed cases. The present findings are limited to clinicopathologic characteristics at diagnosis and organizational aspects of care delivery. Prospective studies with longitudinal follow-up are required to evaluate clinical outcomes and confirm the safety of teledermatology pathways in melanoma management.

In summary, melanomas diagnosed during the teleconsultation period in our tertiary-care setting were characterized by lower Breslow thickness and less frequent regression, and immunosuppression was less common, compared with the 2019 face-to-face cohort. These between-period differences may reflect cohort-level variation in referral pathways and case mix across two structurally distinct care models. Strengthening teledermoscopy availability and standardizing image acquisition in primary care may further improve the performance and safety of teledermatology pathways for suspected melanoma, and prospective multicenter studies are warranted to evaluate diagnostic accuracy, clinical outcomes, and system-level effectiveness.

## 5. Conclusions

In this retrospective single-center comparison of two care models implemented in different periods, melanomas diagnosed through teleconsultation in 2022 showed a more favorable clinicopathologic profile at diagnosis than those diagnosed via face-to-face consultation in 2019, including thinner Breslow thickness and less frequent regression and immunosuppression. Teledermatology-based referral pathways may help streamline access to specialist assessment in tertiary settings; however, causal inference is limited by the observational design and potential differences in referral patterns between periods. Future multicenter studies should evaluate generalizability and assess patient-centered and long-term oncologic outcomes.

## Figures and Tables

**Table 1:** Baseline Demographic, Clinical, Pathological, and Treatment Characteristics of Patients With Melanoma Diagnosed via Face-to-Face Consultation (2019) and Teledermatology (2022).

Characteristic	Face-to-Face 2019 (*n* = 89; 58.9%)	Teleconsultation 2022 (*n* = 62; 41.1%)	Total (*n* = 151, 100%)
**Demographics**			
**Age (mean ± SD), years**	61.14 ± 16.75	59.08 ± 16.01	60.31 ± 16.43
**Sex**			
***Male***	42 (47.2%)	31 (50.0%)	73 (48.3%)
***Female***	47 (52.8%)	31 (50.0%)	81 (53.7%)
**Province of Residence**			
***Granada***	74 (83.1%)	61 (98.4%)	135 (89.4%)
***Almería***	11 (12.4%)	0 (0.0%)	11 (7.3%)
***Jaén***	3 (3.4%)	0 (0.0%)	3 (2%)
***Huelva***	1 (1.1%)	0 (0.0%)	1 (0.7%)
***Córdoba***	0 (0.0%)	1 (1.6%)	1 (0.7%)
**Family and Personal History**			
**Family history of melanoma**			
***No***	85 (95.5%)	56 (90.3%)	141 (93.4%)
***Yes***	4 (4.5%)	6 (9.7%)	10 (6.6%)
**Family history of non-melanoma skin cancer (** **NMSC)**			
***No***	81 (91.0%)	56 (90.3%)	137 (90.7%)
***Yes***	8 (9%)	6 (9.7%)	14 (9.3%)
**Family history of pancreatic cancer**			
***No***	85 (95.5%)	61 (98.4%)	146 (96.7%)
***Yes***	4 (4.5%)	1 (1.6%)	5 (3.3%)
**Personal history of non-melanoma skin cancer (** **NMSC)** **:**			
***No***	83 (93.3%)	57 (91.9%)	140 (92.7%)
***Yes***	6 (6.7%)	5 (8.1%)	11 (7.3%)
**Personal history of melanoma**			
***No***	86 (96.6%)	62 (100.0%)	148 (98.0%)
***Yes***	3 (3.4%)	0 (0.0%)	3 (2.0%)
**Risk Factors**			
**Immunosuppression**			
***No***	81 (91.0%)	61 (98.4%)	145 (94%)
***Yes***	8 (9.0%)	1 (1.6%)	9 (6.0%)
**Fitzpatrick skin phototype**			
***I***	0 (0.0%)	0 (0.0%)	0 (0.0%)
***II***	27 (30.3%)	18 (29.0%)	45 (29.8%)
***III***	60 (67.4%)	43 (69.4%)	103 (68.2%)
***IV***	2 (2.2%)	1 (1.6%)	3 (2.0%)
***V***	0 (0.0%)	0 (0.0%)	0 (0.0%)
***VI***	0 (0.0%)	0 (0.0%)	0 (0.0%)
**Eye colour**			
***Brown***	64 (71.9%)	38 (61.3%)	102 (67.5%)
***Green***	17 (19.1%)	11 (17.7%)	28 (18.5%)
***Blue***	6 (6.7%)	12 (19.4%)	18 (11.9%)
***Black***	2 (2.3%)	1 (1.6%)	3 (2%)
**Hair colour**			
***Black***	1 (1.1%)	0 (0.0%)	1 (0.7%)
***Brown***	45 (50.6%)	35 (56.5%)	80 (53.0%)
***Blond***	37 (41.6%)	24 (38.7%)	61 (40.4%)
***Red***	6 (6.7%)	3 (4.8%)	9 (6.0%)
**Chronic sun exposure**			
***No***	55 (61.8%)	44 (71.0%)	99 (65.6%)
***Yes***	34 (38.2%)	18 (29.0%)	52 (34.4%)
**Intermittent sun exposure**			
***No***	53 (59.6%)	31 (50.0%)	84 (55.6%)
***Yes***	36 (40.4%)	31 (50.0%)	67 (44.4%)
**Childhood sunburns**			
***No***	73 (82.0%)	50 (80.6%)	123 (81.5%)
***Yes***	16 (18.0%)	12 (19.4%)	28 (18.5%)
**Tumor Characteristics**			
**Histopathologic subtype**			
***Superficial spreading melanoma (SSM)***	25 (28.1%)	13 (21.0%)	38 (25.2%)
***Nodular melanoma (NM)***	24 (27.0%)	22 (35.5%)	46 (30.5%)
***Lentigo maligna melanoma (LMM)***	18 (20.2%)	14 (22.6%)	32 (21.2%)
***Acral lentiginous melanoma (ALM)***	1 (1.1%)	2 (3.2%)	3 (2%)
***Desmoplastic melanoma (DM)***	3 (3.4%)	0 (0.0%)	3 (2%)
***Pigmented epithelioid melanocytoma (PEM)***	1 (1.1%)	0 (0.0%)	1 (0.7%)
***Mucosal melanoma***	2 (2.2%)	1 (1.6%)	3 (2%)
***Spitzoid melanoma***	1 (1.1%)	1 (1.6%)	2 (1.3%)
***Others***	12 (13.5%)	9 (14.5%)	21 (13.9%)
**Anatomic site**			
***Head and neck***	16 (18.0%)	10 (16.1%)	26 (17.2%)
***Trunk***	28 (31.5%)	22 (35.5%)	50 (33.1%)
***Upper limbs***	12 (13.5%)	8 (12.9%)	20 (13.2%)
***Lower limbs***	33 (37.1%)	22 (35.5%)	55 (36.4%)
**Histopathologic features**			
**Breslow thickness (mean ± SD), mm**	1.44 ± 1.54	1.20 ± 1.11	1.33 ± 1.37
**Ulceration**			
***Absent***	73 (82.0%)	52 (83.9%)	125 (82.8%)
***Present***	16 (18.0%)	10 (16.1%)	26 (17.2%)
**Mitoses**			
***Absent***	54 (60.7%)	40 (64.5%)	94 (62.3%)
***Present***	35 (39.3%)	22 (35.5%)	57 (37.7%)
**Clark level**			
***I***	0 (0.0%)	0 (0.0%)	0 (0.0%)
***II***	26 (29.2%)	19 (30.6%)	45 (29.8%)
***III***	29 (32.6%)	22 (35.5%)	51 (33.8%)
***IV***	26 (29.2%)	17 (27.4%)	43 (28.5%)
***V***	8 (9.0%)	4 (6.5%)	12 (7.9%)
**Histological Regression**			
***Absent***	56 (62.9%)	44 (71.0%)	102 (66.2%)
***Present***	33 (37.1%)	18 (29.0%)	51 (33.8%)
**Lymphovascular invasion**			
***Absent***	78 (87.6%)	54 (87.1%)	132 (87.4%)
***Present***	11 (12.4%)	8 (12.9%)	19 (12.6%)
**Perineural invasion**			
***Absent***	84 (94.4%)	58 (93.5%)	142 (94.0%)
***Present***	5 (5.6%)	4 (6.5%)	9 (6.0%)
**Tumor size (mean ± SD), cm**	1.27 ± 1.13	1.06 ± 0.74	1.17 ± 0.97
**Treatment and Staging**			
**Sentinel lymph node biopsy performed**			
***No***	58 (65.2%)	38 (61.3%)	96 (63.6%)
***Yes***	31 (34.8%)	24 (38.7%)	55 (36.4%)
**Results of Sentinel lymph node biopsy**			
***Negative***	86 (96.6%)	61 (98.4%)	147 (97.4%)
***Positive***	3 (3.4%)	1 (1.6%)	4 (2.6%)
**AJCC stage**			
***0* (in situ)**	7 (7.9%)	9 (14.5%)	16 (10.6%)
***I***	52 (58.4%)	38 (61.3%)	90 (59.6%)
***II***	15 (16.9%)	8 (12.9%)	23 (15.2%)
***III***	9 (10.1%)	6 (9.7%)	15 (9.9%)
***IV***	2 (2.2%)	1 (1.6%)	3 (2.0%)
**Adjuvant treatment**			
***No***	79 (88.8%)	53 (85.5%)	132 (87.4%)
***Yes***	10 (11.2%)	9 (14.5%)	19 (12.6%)

NMSC: Non-melanoma skin cancer; SSM: Superficial spreading melanoma; NM: Nodular melanoma; LMM: Lentigo maligna melanoma; ALM: Acral lentiginous melanoma; DM: Desmoplastic melanoma; PEM: Pigmented epithelioid melanocytoma.

**Table 2:** Multivariable logistic regression comparing melanomas diagnosed during the teledermatology period (2022) versus the face-to-face period (2019).

Variable	Adjusted OR	95% CI	*p*-Value
Sex	0.80	0.36–1.74	0.568
Age	0.99	0.96–1.02	0.371
Immunosuppression	0.08	0.008–0.86	0.037
Chronic sun exposure	0.53	0.21–1.33	0.176
Intermittent sun exposure	0.33	0.07–1.49	0.150
Childhood sunburns	0.55	0.16–1.83	0.331
Fitzpatrick phototype	1.05	0.46–2.38	0.906
Histopathological subtype	0.89	0.61–1.32	0.565
Anatomic site	0.71	0.46–1.12	0.139
Breslow thickness	0.60	0.40–0.91	0.017
Ulceration	1.32	0.26–6.59	0.737
Mitoses	0.91	0.75–1.10	0.323
Clark level	1.55	0.93–2.57	0.090
Histologic regression	0.28	0.09–0.90	0.032
Lymphovascular invasion	0.21	0.004–10.18	0.428
Perineural invasion	NA	NA	0.987
Diabetes mellitus	0.46	0.14–1.52	0.204
Desmoplastic melanoma	2.77	0.36–21.42	0.330
Tumor size	1.24	0.60–2.52	0.563
Sentinel lymph node biopsy performed	0.89	0.31–2.60	0.837
Sentinel lymph node result	0.67	0.09–4.70	0.686
AJCC stage	1.40	0.62–3.16	0.424

Outcome coded as 0 = face-to-face period (2019) and 1 = teleconsultation period (2022). Adjusted odds ratios (aORs) from multivariable logistic regression are reported with 95% confidence intervals. For variables with sparse data leading to quasi-separation (e.g., perineural invasion), estimates are unstable and CIs are not reported. NA= Not applicable.

## Data Availability

The datasets generated and analyzed during the current study are not publicly available due to patient confidentiality but are available from the corresponding author upon reasonable request.

## Supplementary Materials

The following supporting information can be downloaded at: https://www.mdpi.com/article/10.3390/jcm15010267/s1. File S1: The STROBE checklist.
