# Supplementary material for: Impact of a Teledermatology-Based Referral Model on Melanoma Diagnostic Pathways and Clinicopathologic Features: A Retrospective Comparative Study Between Face-to-Face Consultation (2019) and Teledermatology (2022) in a Tertiary Hospital
Source: J Clin Med. 2025 Dec 29;15(1):267. doi: 10.3390/jcm15010267 (PMC12786944; doi:10.3390/jcm15010267)
Supplement: Supplementary file 1 [file jcm-15-00267-s001.zip › jcm-4053075-supplementary.pdf]

STROBE Checklist for Observational Studies

Title and Abstract

- 1a. Indicate the study design in the title or abstract: Yes (retrospective observational study stated in abstract).
- 1b. Provide an informative and balanced summary: Yes (abstract includes rationale, aim, methods, main results, and conclusion).

Introduction

- 2. Background/rationale: Yes (explains melanoma burden and relevance of teledermatology).
- 3. Objectives: Yes (aims clearly stated).

Methods

- 4. Study design: Yes (retrospective comparative observational study).
- 5. Setting: Yes (tertiary hospital, 2019 F2F vs 2022 teledermatology).
- 6a. Participants: Yes (inclusion/exclusion criteria clearly defined).
- 6b. Matching: Not applicable.
- 7. Variables: Yes (clinical and histopathologic variables defined).
- 8. Data sources/measurement: Yes (electronic medical records and pathology reports).
- 9. Bias: Yes (selection/channeling bias, dermoscopy heterogeneity).
- 10. Study size: Yes (n=151, all eligible cases included).
- 11. Quantitative variables: Yes (continuous variables handled appropriately).
- 12. Statistical methods: Yes (full description of analyses; multivariable logistic regression used).

Results

- 13. Participants: Yes (numbers reported; no follow-up loss).
- 14. Descriptive data: Yes (Table 1; no missing data requiring imputation).
- 15. Outcome data: Yes (coefficients and p-values provided).
- 16. Main results: Yes (adjusted and unadjusted findings; continuous variables kept continuous).
- 17. Other analyses: Yes (univariable and multivariable models in Figures 1–2).

Discussion

- 18. Key results: Yes.
- 19. Limitations: Yes (retrospective design, dermoscopy variability, sample size).
- 20. Interpretation: Yes (balanced and consistent with data).
- 21. Generalisability: Yes (applicable to structured tertiary teledermatology settings).

Other Information

- 22. Funding: No external funding declared.
- Ethics approval: Yes (DERM_HUSC_2024_002).
- Data availability: Available upon reasonable request.
- Conflict of interest: None declared.
